# Care-seeking pathways and diagnostic delays in extrapulmonary TB patients

**DOI:** 10.5588/pha.23.0037

**Published:** 2023-12-07

**Authors:** A. Wali, N. Safdar, A. Ambreen, S. Tahseen, T. Mustafa

**Affiliations:** 1 Centre for International Health, Department of Global Public Health and Primary Care, University of Bergen, Bergen, Norway; 2 Department of Health, Government of Balochistan, Quetta, Pakistan; 3 Interactive Research and Development, Singapore City, Singapore; 4 Department of Microbiology, Gulab Devi Hospital, Lahore, Pakistan; 5 National TB Reference Laboratory, National TB Control Program, Islamabad, Pakistan; 6 Department of Thoracic medicine, Haukeland University Hospital, Bergen, Norway

**Keywords:** Pakistan, EPTB, pleuritis, lymphadenitis, treatment

## Abstract

**SETTING:**

This study was conducted at a private tertiary hospital engaged with the TB control programme in the city of Lahore, Pakistan.

**OBJECTIVE:**

To assess the healthcare-seeking pathways, different delays and factors associated with delays among the patients who presented in the outpatient department with tuberculous lymphadenitis and pleuritis, the most common manifestations of extrapulmonary TB.

**DESIGN:**

This cross-sectional study was conducted prospectively from April 2016 to August 2017.

**RESULTS:**

The median age of the 339 patients analysed was 22 years (IQR 17–30); tuberculous lymphadenitis was predominant in females (63%), while pleuritis affected more males (64%). Overall, 62% reported seeking care from healthcare providers before diagnosis, of whom 62% sought care from private facilities, 32% visited facilities >2 times and 8% visited traditional healers. Diagnostic delay was associated with tuberculous lymphadenitis, age 15–44 years, poor socio-economic status and poor TB knowledge.

**CONCLUSION:**

There was considerable delay in the management of extrapulmonary TB patients, and the health-system delay was the major contributor, leading to increased patient suffering. Efforts towards minimising health-system delay need to be prioritised for patient screening and diagnosis, with a feasible algorithm that is workable in resource-limited settings.

## Abstract (fr)

**CONTEXTE:**

Cette étude a été menée dans un hôpital tertiaire privé participant au programme de lutte contre la TB dans la ville de Lahore, au Pakistan.

**OBJECTIF:**

Évaluer les parcours de recherche de soins, les différents délais et les facteurs associés aux délais parmi les patients qui se sont présentés au service des consultations externes avec une lymphadénite et une pleurite tuberculeuses, les manifestations les plus courantes de la TB extrapulmonaire.

**MÉTHODE:**

Cette étude transversale a été menée prospectivement d’avril 2016 à août 2017.

**RÉSULTATS:**

L’âge médian des 339 patients analysés était de 22 ans (IQR 17–30) ; la lymphadénite tuberculeuse prédominait chez les femmes (63%), tandis que la pleurite touchait davantage les hommes (64%). Dans l’ensemble, 62% des patients ont déclaré avoir eu recours à des prestataires de soins de santé avant le diagnostic, dont 62% dans des établissements privés, 32% ont visité des établissements >2 fois et 8% ont consulté des guérisseurs traditionnels. Le retard de diagnostic était associé à la lymphadénite tuberculeuse, à l’âge de 15 à 44 ans, à un statut socio-économique défavorable et à un manque de connaissances sur la TB.

**CONCLUSION:**

La prise en charge des patients atteints de TB extrapulmonaire accuse un retard considérable, dont le système de santé est le principal responsable, ce qui accroît les souffrances des patients. Les efforts visant à minimiser le retard du système de santé doivent être priorisés pour le dépistage et le diagnostic des patients, avec un algorithme réalisable dans des environnements aux ressources limitées.

TB remains a major public health problem worldwide, and one of the leading causes of death from a single infectious agent. TB mainly affects the lungs; when it affects any other body organ, it is called extrapulmonary TB (EPTB), which accounts for roughly 20–30% of active TB cases.^1^–^3^ In 2021, 6.4 million TB cases were notified out of an estimated 10.6 million incidents, and among those notified, 18% were EPTB.^4^ As per public health perception, EPTB is rarely infectious; these cases are therefore given a low priority in high-burden settings, and many cases are suspected to remain undetected.^5^–^7^ Healthcare-seeking challenges cause diagnostic delays, drug resistance, and an increase in morbidity and mortality.^8^–^11^ Globally, studies on healthcare-seeking pathways and diagnostic delays in TB patients are focused mainly on pulmonary TB (PTB), and limited information is available on EPTB.^12^,^13^

In Pakistan, an important challenge for TB control is delayed case detection, and inadequate diagnosis and treatment.^14^ Despite enhanced TB control efforts, a large proportion of TB patients are still being managed outside national TB guidelines.^11^ Understanding healthcare-seeking pathways from symptom onset to the final diagnosis of EPTB and its outcome, including the way patients interact with the healthcare system, is required to improve EPTB care in high-burden settings like Pakistan. This study aimed to assess healthcare-seeking pathways among the patients who presented to the outpatient department with tuberculous lymphadenitis (TBLN; the most common manifestation of EPTB) and tuberculous pleuritis (TBP; the second most prevalent form of EPTB), constituting 35–40% of all EPTB cases,^15^–^17^ and the factors associated with the diagnostic and treatment delays in a tertiary-level hospital in Pakistan.

## METHODS

### Study design

This cross-sectional study was part of a more extensive study designed to evaluate the implementation of a new diagnostic, the MPT64 antigen detection test, for the diagnosis of EPTB.^15^,^16^

### General setting

Pakistan is a high TB burden country, and its share of 6% among the top eight countries accounts for two-thirds of the 10 million new estimated global TB cases.^17^ In 2021, it was estimated that there would be about 611,000 new TB cases; however, only 339,300 TB cases were reported, of which 61,074 (18%) were EPTB cases.^4^ The decentralised public health system and a largely unregulated private sector comprising qualified and unqualified service providers deliver healthcare services, including TB care, to the population in Pakistan.^18^ The present study was conducted at the Gulab Devi Hospital (GDH), a not-for-profit private tertiary care hospital in the city of Lahore, Pakistan.

### Study population

The study participants were enrolled prospectively from April 2016 to August 2017. Patients of any age from any gender who presented in the outpatient department with presumed tuberculous lymphadenitis (TBLN) and tuberculous pleuritis (TBP) and investigated, diagnosed and registered for anti-TB treatment (ATT) at GDH were included in the study. The patients who had not given informed written consent and were referred for treatment to a TB care facility near their residence were excluded from the study, as they could not be followed up for final treatment outcomes.

### Data collection and variables

Data were collected by interviewing study participants using a pre-designed questionnaire adapted from a multicentre study.^19^ The questionnaire was translated into Urdu, the national language. Parents or guardians of study participants under the age of 15 signed the written consent and answered the interviewer’s questions. The patients were diagnosed and treated by the physician, while two nurses (research assistants) were responsible for following the patients, dispensing drugs, completing research questionnaires, and collecting blood samples. The distance between the patient’s residence and the health facility was measured using Google Maps (Google, Mountain View, CA, USA) in order to estimate transport costs. To note, most patients were from Lahore City and its vicinity, which has a good road network, good public transport connections and uniform traffic problems. Clinical and diagnostic workup data were obtained from the patients’ personal medical record file. Some participants avoided answering the questions on smoking status and chewable tobacco use due to social conventions; these were recorded as ‘not applicable’. Data on the routine diagnostic workup were acquired from the laboratory, encompassing acid-fast bacilli staining, culture, Xpert® MTB/RIF assay (Cepheid, Sunnyvale, CA, USA), cytology and histology results, as well as complete blood count, erythrocyte sedimentation rate, and relevant radiological examinations.

### Operational definitions

Patient delay was defined as the period from symptom onset to the date of first contact with the healthcare provider in days. The health system delay was defined as the interval between the first visit to a healthcare provider and the date of enrolment for ATT for diagnosed EPTB disease. Total delay is the sum of the periods from symptom onset to the date of enrolment for ATT. For analysis purposes, patients were categorised using a composite reference standard (CRS), which was devised based on clinical signs and symptoms, radiological findings, results from laboratory investigations, response to specific non-TB therapy and response to ATT.

### Data analysis and statistics

Data were double entered, validated, and analysed using EpiData v3.1 for entry and v2.2.2.183 for review (EpiData Association, Odense, Denmark). Descriptive statistics were presented, and group differences were compared using the χ^2^ test for proportions and non-parametric tests for numeric variables. Bonferroni adjustments were made for the multiple tests. Kuppuswamy’s socio-economic status scale was used to assess socio-economic status.^20^,^21^ Composite knowledge about TB and EPTB was determined based on responses to four separate knowledge elements with multiple questions.^22^

### Ethics approval and consent to participate

Ethical clearance was obtained from the Regional Committee for Medical and Health Research Ethics, Western Norway (REK Vest), Oslo, Norway; and the National Bioethics Committee Pakistan, Islamabad, Pakistan. All study participants provided informed written consent. For participants under the age of 15 years, written consent was provided by the parent or guardian.

## RESULTS

Out of 390 presumptive EPTB patients, 339 (87%) were categorized by CRS and analysed in the study. Of the enrolled patients, 18% were children (3–14 years) and 82% were adults, and among adults, most of the patients (87%) were between 15–44 years of age. TB lymphadenitis was more prevalent (63%) in females, while TB pleuritis affected more (64%) males. About 87% of patients were non-smokers, and 91% did not use chewable tobacco Table 1.

**TABLE 1:** Sociodemographic characteristics of tuberculous lymphadenitis and pleuritis patients seeking healthcare at GDH, Lahore, Pakistan (*n* = 339)

Characteristics	Total *n* (%)	TB lymphadenitis (*n* =187) *n* (%)	TB pleuritis (*n* =152) *n* (%)	*P* value*
Age groups, years				0.00
3–14	60 (18)	56 (30)	4 (3)	
15–44	244 (72)	122 (65)	122 (80)	
≥45	35 (10)	9 (5)	26 (17)	
Sex				0.00
Male	167 (49)	70 (37)	97 (64)	
Female	172 (51)	117 (63)	55 (36)	
Smoking status				0.00
Yes	36 (11)	10 (5)	26 (17)	
No	245 (72)	137 (73)	108 (71)	
NA^†^	58 (17)	40 (21)	18 (12)	
Chewable tobacco use				0.00
Yes	24 (7)	8 (5)	16 (11)	
No	258 (76)	139 (74)	119 (78)	
NA^†^	57 (17)	40 (21)	17 (11)	
SES class				0.54
Very poor	16 (5)	8 (4)	8 (5)	
Poor	293 (86)	165 (88)	128 (84)	
Middle	30 (9)	14 (8)	16 (11)	
Place of residence				0.49
Within city	223 (66)	126 (67)	97 (64)	
Out of city	116 (34)	61 (33)	55 (36)	
Distance between home and GDH, km				0.47
0–50	241 (71)	138 (74)	103 (68)	
51–300	92 (27)	46 (24)	46 (30)	
≥301	6 (2)	3 (2)	3 (2)	
Travel time GDH, min				0.48
≤60	19 (6)	9 (5)	10 (7)	
>60	320 (94)	178 (95)	142 (93)	
Household size, *n*				0.05
1–5	91 (27)	60 (32)	31 (20)	
6–10	179 (53)	93 (50)	86 (57)	
≥11	69 (20)	34 (18)	35 (23)	

GDH = Gulab Devi Hospital; SES = socio-economic status; NA = not applicable.

*Comparing group differences of TB lymphadenitis and pleuritis, *P*-value ≤ 0.05 (significant).

^†^Patients avoided answering the questions due to social conventions, recorded as ‘Not applicable’.

The most common systemic symptoms were fever (87%), followed by appetite loss (54%) and weight loss (57%). Neck mass (47%), difficulty in breathing (38%) and chest pain (36%) were the predominant local symptoms (Table 2). The median composite TB knowledge score was 2 (IQR 0–2), and the majority (80%) had poor knowledge about TB.

**TABLE 2:** Symptoms and their durations of tuberculous lymphadenitis and pleuritis patients prior to presentation for healthcare seeking at Gulab Devi Hospital, Lahore, Pakistan

Symptoms and durations of symptoms	TB lymphadenitis	*P* value^†^	TB pleuritis	*P* value^†^
	*n/N* (%)*		*n/N* (%)*	
Fever				
Yes	123/148 (83)	0.04	123/135 (91)	0.04
No	25/148 (17)		12/135 (9)	
Duration of fever, weeks				
>4	82/108 (76)	0.12	80/120 (33)	0.12
1–4^‡^	26/108 (24)		40/120 (67)	
Appetite loss				
Yes	57/146 (39)	0.00	95/136 (70)	0.00
No	89/146 (61)		41/136 (30)	
Duration of appetite loss, weeks				
>4	45/54 (83)	0.00	58/95 (61)	0.00
1–4^‡^	9/54 (17)		37/95 (39)	
Weight loss				
Yes	72/150 (48)	0.00	89/135 (66)	0.00
No	78/150 (52)		46/135 (34)	
Duration of weight loss, weeks				
>4	49/52 (94)	0.26	61/69 (88)	0.26
1–4^‡^	3/52 (6)		8/69 (12)	
Night sweats				
Yes	17/143 (12)	0.00	40/129 (31)	0.00
No	126/143 (88)		89/129 (69)	
Duration of night sweats, weeks				
>4	14/16 (88)	0.03	21/37 (57)	0.03
1–4^‡^	2/16 (12)		16/37 (43)	
Neck mass				
Yes	121/143 (85)	0.00	—	
No	22/143 (15)		—	
Duration, weeks				
>16	108/117 (92)	0.03	—	
1–16^‡^	9/117 (8)		—	
Breathing difficulty				
Yes	—		82/135 (61)	0.00
No	—		53/135 (39)	
Duration of breathing difficulty, weeks				
>2	—		69/77 (90)	0.59
1–2^‡^	—		8/77 (10)	
Chest pain				
Yes	—		83/135 (62)	0.00
No	—		52/135 (38)	
Duration of chest pain, weeks				
>2	—		10/80 (12)	0.17
1–2^‡^	—		70/80 (88)	

*Number of patients for whom data were not available were excluded from the analysis.

^†^Comparing group differences of TB lymphadenitis and TB pleuritis, *P* < 0.05 (significant).

^‡^Median value for symptom duration used to dichotomise duration in weeks.

**FIGURE 1:**
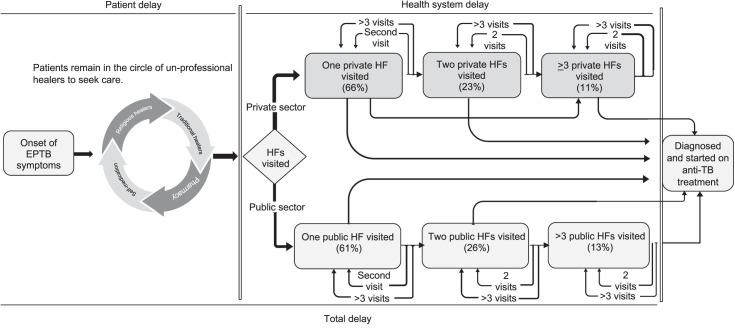
Healthcare-seeking pathways of tuberculous lymphadenitis and pleuritis patients at Gulab Devi Private Hospital, Lahore, Pakistan. Patients continue to visit unprofessional healers to seek treatment before entering a formal professional healthcare system (indicated by the circle with arrows). The number of patients moving in the direction of the arrow in the private healthcare system is shown in the upper level boxes in light grey, while the number of patients moving in the direction of the arrow in the public healthcare system is shown in the lower boxes. The size of the inverted arrows corresponds to the number of patients who visited each site for the first and subsequent visits at the same HF in the relevant sector (private or public) of the healthcare system before reaching the study site. EPTB = extrapulmonary TB; HF = healthcare facility.

Overall, 209 (62%) reported seeking care from healthcare providers prior to an EPTB diagnosis, 130 (62%) of whom sought care from private health facilities and 71 (32%) visited health facilities >2 times, while 16 (8%) visited traditional healers, sometimes several times (Figure 1).

**FIGURE 2:**
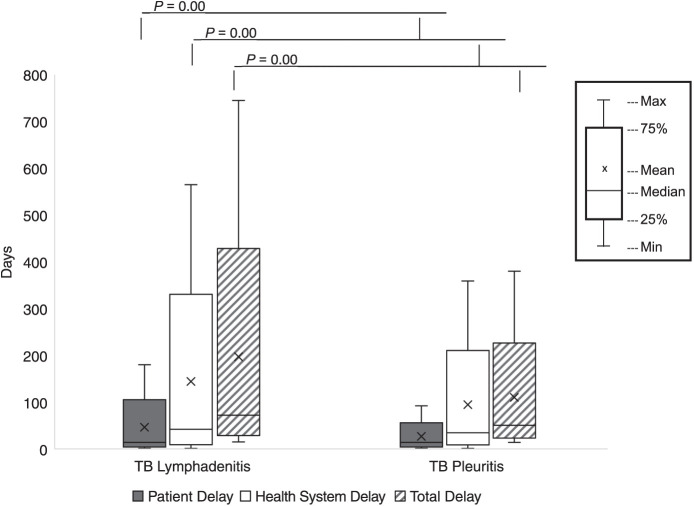
Box plot showing the distribution of patient, health-system and total delays among patients with tuberculous lymphadenitis and pleuritis. Significant differences in patient, health system and total delays were observed based on Mann–Whitney’s test. When comparing group variances between TB lymphadenitis (*n* = 187) and TB pleuritis (*n* = 152), all *P*-values were ,0.05. Error bars reflect the 25^th^ (lower error bar) and 75^th^ (upper error bar) quartiles, and data are shown as median and interquartile ranges.

Overall, patients with TB lymphadenitis had longer delays than those with TB pleuritis. Among TB lymphadenitis patients, median patient, health system delay and total delays were respectively 14 days (IQR 7–30), 42 days (IQR 17–96) and 72 days (IQR 42–112). In TB pleuritis, median patient, health system and total delays were respectively 14 days (IQR 7–20), 34 days (IQR 16–72) and 50 days (IQR 32–73). About 9% of patients had a total delay of more than 6 months, and 1% had a total delay of more than 1 year. Among the patients delayed more than 6 months and more than 1 year, respectively 78% and 77% were diagnosed with TB lymphadenitis. Health system delay was significantly longer than patient delay (*P* < 0.05), and was the biggest contributor to the total delay. The distribution of different delays according to the disease sites is given in Figure 2.

TB lymphadenitis, poor socio-economic class and poor TB knowledge were associated with prolonged health system and total delays (*P* < 0.05). In addition, total delay was greater among patients aged 15–44 years (*P* < 0.05; Table 3). TB lymphadenitis patients experiencing a neck mass symptom duration of >16 weeks and TB pleuritis patients experiencing a symptom duration of >4 weeks experienced 2.13 times more likely to experience prolonged patient delays of >14 days. (Supplementary Table S1). Median healthcare system delay in both lymphadenitis (42 days) and pleuritis (34 days) patients was longer than patient delay (>14 days).

**TABLE 3:** Factors associated with the patient and health system delays among tuberculous lymphadenitis and pleuritis patients seeking care at Gulab Devi Hospital Lahore, Pakistan (*n* = 339)

Characteristics	Patient delay (≥14 days)		Health system delay (≥40 days)		Treatment delay (≥69 days)	
	OR (95% CI)	*P-*value*	OR (95% CI)	*P-*value*	OR (95% CI)	*P-*value*
EPTB infection site						
Lymphadenitis	1.37 (0.88–2.12)	0.16	1.62 (1.06–2.50)	0.02	1.86 (1.21–2.87)	0.00
Pleuritis	1^†^		1^†^		1^†^	
Age groups, years						
0–14	1^†^		1^†^		NA	
15–44	1.54 (0.91–2.62)	0.10	1.59 (0.96–2.63)	0.07	1.94 (1.17–3.23)	0.01
≥45	0.80 (0.17–3.67)	0.77	1.47 (0.30–7.19)		1^†^	
Sex						
Female	1.13 (0.73–1.75)	0.58	1.19 (0.78–1.83)	0.41	1.44 (0.94–2.21)	0.09
Male	1^†^		1^†^		1^†^	
SES class						
Middle	1.60 (0.24–10.81)	0.62	1.60 (0.24–10.81)	0.60	1.31 (0.19–9.10)	0.78
Poor	1.44 (0.86–2.39)	0.16	1.90 (1.15–3.14)	0.01	2.48 (1.49–4.13)	0.00
Very poor	1^†^		1^†^		1^†^	
Place of residence						
Out of city	1.01 (0.63–1.60)	0.97	0.91 (0.58–1.42)	0.67	1.04 (0.66–1.63)	0.85
Within city	1^†^		1^†^		1^†^	
First visited HCF						
Private	1.23 (0.67–2.27)	0.51	0.95 (0.51–1.77)	0.86	1.10 (0.59–2.03)	0.77
Public	1^†^		1^†^		1^†^	
Travel time to HCF, min						
≥60	0.95 (0.36–2.47)	0.91	1.45 (0.57–3.69)	0.43	1.92 (0.74–5.00)	0.17
<60	1^†^		1^†^		1^†^	
Healthcare contacts‡						
Single	1.06 (0.58–1.93)	0.85	1.06 (0.58–1.95)	0.84	0.96 (0.53–1.76)	0.90
Multiple	1^†^		1^†^		1^†^	
Self–medication						
Yes	1.07 (0.64–1.77)	0.80	1.46 (0.88–2.42)	0.13	1.28 (0.78–2.11)	0.33
No	1^†^		1^†^		1^†^	
TB knowledge^§^						
Poor	1.20 (0.67–2.15)	0.54	1.84 (1.10–3.08)	0.01	1.75 (1.05–2.95)	0.03
Good	1^†^		1^†^		1^†^	

*Total number of visits to health facilities and number of health facilities.

^†^Comparing group differences in patient, health system and total delays, *P* < 0.05 significant.

^‡^Numbers of contacts (visits) to healthcare facilities.

^§^Composite knowledge scores about TB was created from four individual knowledge items; median of composite score used to dichotomise knowledge as Poor = 1–2 and Good = 3–4.

EPTB = extrapulmonary TB; OR = odd ratio; CI = confidence interval; SES = socio-economic status; HCF = healthcare facility.

## DISCUSSION

Healthcare system delays were reported to be longer than patient delays by a number of studies, while some other studies have found the opposite.^11^,^23^,^24^ Studies documented longer health system delays among EPTB patients than those with PTB.^25^,^26^ Prolonged healthcare system delays may be due to the fact that healthcare providers did not initiate specific EPTB investigation regardless of typical systemic, as well as local symptoms such as fever, appetite loss, weight loss, night sweats, neck mass, breathing difficulty and chest pain. EPTB investigation and treatment were initiated only after multiple visits to multiple health facilities.^25^ A study from India supports our study findings, which reported that the main reason for longer health system delays (42 days) was the failure to prescribe the correct EPTB diagnostic investigation by healthcare providers at first contact,^23^ even when patients presented with typical symptoms. In contrast to our findings, two studies conducted in Ethiopia attributed the delayed diagnosis of EPTB to prolonged patient delay, which was primarily associated with a lack of recognition of EPTB symptoms and not taking these seriously, resulting in patients not seeking healthcare at a suitable health facility.^26^,^27^ In order to prevent both patient and health system delays, it is necessary to increase the capacity of healthcare providers through training programmes and an emphasis on community awareness about EPTB.

We are not aware of any consensus on what would constitute an acceptable delay in the diagnosis and treatment initiation in EPTB patients. Several investigators have deemed a patient delay of ≥14 days as unacceptable and related it to an increased need for transfer to a tertiary-level TB hospital; to note, a health system delay of ≥30 days was associated with higher mortality.^14^,^27^ If this cut-off is applied to our study data, the median health system delay of 40 days was higher than the acceptable delay, and only 39% had an acceptable delay time, and only 38% of the patients contacted the health facility within the acceptable patient delay time. Patients with TB lymphadenitis experienced the longest total delay of 72 days (IQR 42–112) compared to 51 days (IQR 32–73) for TB pleuritis patients. Previous studies from Zanzibar and India also showed a longer median total delay of respectively 99 and 84 days among TB lymphadenitis patients, compared to 34 and 28 days among TB pleuritis and other sites of the EPTB disease.^19^,^23^ Several contributing factors may explain the longer delays, such as the enormous diversity of clinical presentations of EPTB disease, the complexity of the usual diagnostic investigations, the need for multiple confirmative investigations, including time-consuming mycobacterial culture, the lack of adequate laboratory services at peripheral health facilities, the extraordinary cost of the investigations and equipment, the need for surgical invasive procedures and the lack of skilled staff for carrying out invasive procedures and sample collection.^11^,^19^ The healthcare system needs resources for devising screening algorithms, skilled human resource development, and rapid and more sensitive diagnostic tools to standardise EPTB care.

A significantly higher number of patients with TB pleuritis had both systemic and local symptoms than those with TB lymphadenitis. There was no difference in the number of patients with TB pleuritis based on the duration of symptoms. However, among TB lymphadenitis patients, a larger proportion presented with a longer duration of symptoms, indicating that these patients tended to delay seeking healthcare for a more extended period of time.^28^ Taken together, these findings indicate that TB pleuritis follows a relatively more acute or sub-acute course, as corroborated by other studies,^29^–^32^ while patients with TB lymphadenitis are more likely to be indolent, resulting in delays in seeking healthcare.^33^,^34^ The primary local symptom reported by 85% of patients with TB lymphadenitis was a neck mass. This finding contrasts with a report from India, where a lower percentage of patients reported neck masses as a symptom.^23^

Our aim was to study the symptoms that would prompt patients to seek care earlier. Our results indicate that patients with symptoms such as fever, weight loss, night sweats, breathing difficulty and chest pain did not delay seeking care, while those with neck mass and loss of appetite were less likely to contact a healthcare facility early. Both patients and healthcare professionals appeared to ignore persistent, indolent symptoms, which can accelerate disease progression, leading to increased complications, poor treatment outcomes and mortality. Raising awareness about the need for early detection of EPTB symptoms among healthcare providers, as well as patients, could lead to timely and appropriate management.

Our study had some strengths. First, we did not exclude any patient based on age, as this study was part of a bigger project aimed at obtaining an overview of EPTB in different age groups, including children, as factors associated with delays in diagnosis and treatment and the healthcare-seeking pathway could vary according to age group. Second, our study design provided better logistic measures, i.e., study design was based on a pre-tested systematic data collection tool (questionnaire). Third, the study mostly used available resources in collaboration with TB control programmes.

Our study had some limitations. Patients were only enrolled at a single tertiary-level private hospital, and patients who began treatment at other private or public healthcare facilities were not included in the study. Results may thus not be generalisable to all EPTB patients presenting to the healthcare system in Pakistan. Information about symptoms, symptom onset and healthcare-seeking activities were mostly self-reported and may have led to recall bias.

## CONCLUSION

The result of this study indicates that most patients with TB lymphadenitis, as well as those with TB pleuritis experienced diagnostic and treatment delays beyond 2 months, mostly due to health system delays. A decline in health system delays, ensuring adequate diagnosis and timely treatment, could be achieved by strengthening presumptive EPTB case identification and the establishment of an active referral network among peripheral TB care facilities with facilities for diagnostic tools, sample collection, and the recruitment of knowledgeable and skilled healthcare providers.

## References

[1] Golden MP. (2005). Extrapulmonary tuberculosis: an overview. Am Fam Physician.

[2] Hoel IM (2020). Diagnosis of extrapulmonary tuberculosis using the MPT64 antigen detection test in a high-income low tuberculosis prevalence setting. BMC Infect Dis.

[3] Lee JY. (2015). Diagnosis and treatment of extrapulmonary tuberculosis. Korean Natl Tuberc Assoc.

[4] World Health Organization (2022). Global tuberculosis report, 2022.

[5] Gomes T (2014). Epidemiology of extrapulmonary tuberculosis in Brazil: a hierarchical model. BMC Infect Dis.

[6] Tandirogang N (2020). The spatial analysis of extrapulmonary tuberculosis spreading and its interactions with pulmonary tuberculosis in Samarinda, East Kalimantan, Indonesia.

[7] Tahseen S (2020). Extrapulmonary tuberculosis in Pakistan- A nation-wide multicenter retrospective study. PLoS One.

[8] Tesgaye F, Defar A, Beyene T. (2014). Documentation and treatment outcomes of smear-negative and extra-pulmonary tuberculosis in Ethiopia. Int Tuberc Against Dis Lung.

[9] Rehman S (2018). Pattern diagnosis and treatment outcome of extra pulmonary tuberculosis. Pak J Chest Med.

[10] Whitehorn J, Ayles H, Godfrey-Faussett P. (2010). Extra-pulmonary and smear-negative forms of tuberculosis are associated with treatment delay and hospitalisation. Int J Tuberc Lung Dis.

[11] Purohit MR, Purohit R, Mustafa T. (2019). Patient health seeking and diagnostic delay in extrapulmonary tuberculosis: a hospital based study from Central India. Tuberc Res Treat.

[12] Nyatichi FO, Amimo FA. (2016). Factors contributing to delay in seeking treatment among pulmonary tuberculosis patients in Suneka sub-county Kenya. J Heal Educ Res Dev.

[13] Said K (2017). Diagnostic delay and associated factors among patients with pulmonary tuberculosis in Dar es Salaam, Tanzania. Infect Dis Poverty.

[14] Pakistan National TB Control Programme (2019). National guidelines for the control of tuberculosis in Pakistan.

[15] Zhai K, Lu Y, Shi HZ. (2016). Tuberculous pleural effusion. J Thorac Dis.

[16] Tadele A (2014). Immunocytochemical detection of *Mycobacterium tuberculosis* complex specific antigen, MPT64, improves diagnosis of tuberculous lymphadenitis and tuberculous pleuritis. BMC Infect Dis.

[17] Gopalaswamy R (2021). Extrapulmonary tuberculosis—an update on the diagnosis, treatment and drug resistance. J Respir.

[18] Jørstad MD (2018). MPT64 antigen detection test improves routine diagnosis of extrapulmonary tuberculosis in a low-resource setting: a study from the tertiary care hospital in Zanzibar. PLoS One.

[19] Tahseen S (2019). Primary drug resistance in extra-pulmonary tuberculosis: A hospital-based prospective study from Pakistan. Int J Tuberc Lung Dis.

[20] Abou Jaoude GJ (2022). National tuberculosis spending efficiency and its associated factors in 121 low-income and middle-income countries, 2010–19: a data envelopment and stochastic frontier analysis. Lancet Glob Heal.

[21] Yaqoob A (2021). Diagnosis of childhood tuberculosis in Pakistan: are national guidelines used by private healthcare providers?. Int J Infect Dis.

[22] Jørstad MD (2018). Diagnostic delay in extrapulmonary tuberculosis and impact on patient morbidity: a study from Zanzibar. PLoS One.

[23] Mughal AR (2011). Socioeconomic status and impact of treatment on families of children with congenital heart disease. J Coll Physicians Surg Pakistan.

[24] Saleem SM, Jan SS. (2021). Modified Kuppuswamy socioeconomic scale updated for the year 2021. Indian J Forensic Community Med.

[25] Naidoo P (2016). Predictors of knowledge about tuberculosis: Results from SANHANES I, a national, cross-sectional household survey in South Africa. BMC Public Health.

[26] Grover M (2014). Treatment pathways of extrapulmonary patients diagnosed at a tertiary care hospital in Delhi, India. Lung India.

[27] Lienhardt C (2001). Factors affecting time delay to treatment in a tuberculosis control programme in a sub-Saharan African country: the experience of The Gambia. Int J Tuberc Lung Dis.

[28] Seid A, Metaferia Y. (2018). Factors associated with treatment delay among newly diagnosed tuberculosis patients in Dessie city and surroundings, Northern Central Ethiopia: a cross-sectional study. BMC Public Health.

[29] Tattevin P (2012). Factors associated with patient and health care system delay in the diagnosis of tuberculosis in France. Int J Tuberc Lung Dis.

[30] Belay M (2012). Diagnostic and treatment delay among tuberculosis patients in Afar Region, Ethiopia: a cross-sectional study. BMC Public Health.

[31] Bilchut AH, Mekonnen AG, Assen TA. (2022). Knowledge of symptoms and delays in diagnosis of extrapulmonary tuberculosis patients in North Shewa zone, Ethiopia. PLoS One.

[32] Shaw JA, Diacon AH, Koegelenberg CFN. (2019). Tuberculous pleural effusion. Respirology.

[33] Mesfin MM (2006). Delays and care seeking behavior among tuberculosis patients in Tigray of Northern Ethiopia. Ethiop J Heal Dev.

[34] de Almeida CPB, Skupien EC, Silva DR. (2015). Health care seeking behavior and patient delay in tuberculosis diagnosis. Cad Saude Publica.

