# Supplementary material for: Care-seeking pathways and diagnostic delays in extrapulmonary TB patients
Source: Public Health Action. 2023 Dec 7;13(4):148–54. doi: 10.5588/pha.23.0037 (PMC10703130; doi:10.5588/pha.23.0037)
Supplement: Supplementary file 1 [file iutld_pha_23.0037_supplementarydata1.pdf]

http://dx.doi.org/10.5588/pha.23.0037

Care-seeking pathways and diagnostic delays in extrapulmonary TB patients

Supplementary Table S1. Association of symptom's duration with prolong (≥14 days) patients delay of tuberculous lymphadenitis and pleuritis patients.

Symptom's durations	TB lymphadenitis		TB pleuritis	
	OR (95 % CI)	p-value[1]	OR (95 % CI)	p-value[1]
Fever				
1-4 weeks[2]	1		1	
>4 weeks	1.71 (0.92-3.18)	0.08	1.49 (0.78-2.85)	0.22
Appetite loss				
1-4 weeks[2]	1		1	
>4 weeks	1.90 (0.89-4.05)	0.09	2.13 (1.07-4.24)	0.02
Weight loss				
1-4 weeks[2]	1		1	
>4 weeks	0.99 (0.47-2.05)	0.97	1.52 (0.73-3.17)	0.25
Night sweats				
1-4 weeks[2]	1		1	
>4 weeks	0.96 (0.31-2.98)	0.93	1.54 (0.58-4.07)	0.38
Neck mass				
1-16 weeks[2]	1		-	
>16 weeks	2.13 (1.09-4.16)	0.02	-	
Breathing difficulty				
1-2 weeks[2]	-		1	
>2 weeks	-		1.12 (0.59-2.14)	0.72
Chest pain				
1-2 weeks[2]	-		1	
>2 weeks	-		0.94 (0.50-1.80)	0.86

TB, tuberculosis; OR, Odd ratio; CI, Confidence interval; status.

[1] Comparing group differences of symptoms duration in weeks, p-value <0.05 significant.

[2] Median of the symptoms duration used to dichotomize the duration in weeks.

1: Reference category.
